# Synthesizing realistic high-resolution retina image by style-based generative adversarial network and its utilization

**DOI:** 10.1038/s41598-022-20698-3

**Published:** 2022-10-15

**Authors:** Mingyu Kim, You Na Kim, Miso Jang, Jeongeun Hwang, Hong-Kyu Kim, Sang Chul Yoon, Yoon Jeon Kim, Namkug Kim

**Affiliations:** 1 Department of Convergence Medicine, University of Ulsan College of Medicine, Asan Medical Center, 88 Olympic-ro 43-gil, Songpa-gu, Seoul, 05505 Republic of Korea; 2 Department of Ophthalmology, University of Ulsan College of Medicine, Asan Medical Center, 88 Olympic-ro 43-gil, Songpa-gu, Seoul, 05505 Republic of Korea; 3 Department of Biomedical Engineering, Asan Medical Institute of Convergence Science and Technology, University of Ulsan College of Medicine, Asan Medical Center, Seoul, Republic of Korea; 4 Division of Medical Oncology, Department of Internal Medicine, Korea University College of Medicine, Seoul, Republic of Korea; 5 Department of Biomedical Research Center, Korea University Guro Hospital, Seoul, Republic of Korea; 6 Department of Health Screening and Promotion Center, Asan Medical Center, Seoul, Republic of Korea; 7 Department of Ophthalmology, Yonsei University College of Medicine, Seoul, Republic of Korea; 8 Department of Radiology, University of Ulsan College of Medicine, Asan Medical Center, Seoul, Republic of Korea

**Keywords:** Medical research, Biomedical engineering, Medical imaging

## Abstract

Realistic image synthesis based on deep learning is an invaluable technique for developing high-performance computer aided diagnosis systems while protecting patient privacy. However, training a generative adversarial network (GAN) for image synthesis remains challenging because of the large amounts of data required for training various kinds of image features. This study aims to synthesize retinal images indistinguishable from real images and evaluate the efficacy of the synthesized images having a specific disease for augmenting class imbalanced datasets. The synthesized images were validated via image Turing tests, qualitative analysis by retinal specialists, and quantitative analyses on amounts and signal-to-noise ratios of vessels. The efficacy of synthesized images was verified by deep learning-based classification performance. Turing test shows that accuracy, sensitivity, and specificity of 54.0 ± 12.3%, 71.1 ± 18.8%, and 36.9 ± 25.5%, respectively. Here, sensitivity represents correctness to find real images among real datasets. Vessel amounts and average SNR comparisons show 0.43% and 1.5% difference between real and synthesized images. The classification performance after augmenting synthesized images outperforms every ratio of imbalanced real datasets. Our study shows the realistic retina images were successfully generated with insignificant differences between the real and synthesized images and shows great potential for practical applications.

## Introduction

Owing to the rapid development of computer vision with the help of deep learning, many researchers have attempted to develop artificial-intelligence (AI)-based computer-aided diagnosis (CAD) systems for medical fields^1,2^. AI-based CAD systems show significant potential to increase the accuracy of diagnoses and enable appropriate treatment plans based on the predicted disease progression. Recently, retinal images are also being actively studied for the diagnosis of various ocular diseases such as diabetic retinopathy (DR)^3,4^, age-related macular disease (AMD)^5,6^, and glaucoma^7,8^.

Because of the various features in retinal images that need to be learned, developing a robust interpretation tool for retinal diseases remains challenging. The best approach to learn the various features of retinal images is to collect big data that are sufficiently large to cover all disease patterns and patient-dependent variations, including distributions of race, ethnicity, age, and sex^9,10^. Recently, increasing demand for medical care and advances in medical technology gradually enable to collect images covering those features^11^. On the other hand, the AI-based generation model demonstrated the synthesized image has efficacy for balancing imbalanced datasets for diagnostic model development with avoidance from patient privacy issues^12^. Generative adversarial Network (GAN) is a powerful deep learning algorithm that synthesizes high-resolution images in an unsupervised manner^13^. GAN is composed of two deep-learning networks: a generator, which attempts to synthesize realistic images, and a discriminator, which learns image characteristics from training data and attempts to discriminate whether the synthesized images are real or synthesized. The discriminator feedbacks its answer (i.e., degree of realism defined with similarity metric) to the generator via backpropagation, to enable the generator to modify its weight and synthesize more realistic images. Several studies on synthesizing retinal images via GAN have already been conducted^14–20^. Nevertheless, for most of these studies, the spatial resolutions of the synthesized images were poor, and the small numbers of trained images resulted in blurred and low-contrast optic discs, vessels, and retina boundaries. In addition, most of these previous studies concentrated on the textures and landmarks of retinal images by maintaining the vascular structures.

To overcome these problems, we used large and high-resolution datasets for training our GAN to synthesize high-resolution retinal images that are indistinguishable from real images. The synthesized images were evaluated using three kinds of methods: an image Turing test, qualitative evaluation by retinal specialist and comparisons of an amount and a signal-to-noise ratio of the vessels. After evaluations of synthesized images, retinal images having only specific disease were retrained by transfer learning the weight trained with large and high-resolution datasets. Through this, the efficacy of the transfer learning was verified even using the small number of images compared with minimum number of images that empirically known^21^. The verification of transfer learning was done via a classification task by adding synthesized disease images to the imbalanced real dataset.

This paper contributes to the literature that developed using large amounts of high-resolution data and therefore delivered realistic high-resolution images. In addition, we showed synthesizing the retinal images having disease can be possible by transfer learning the weight trained from normal data although disease cases usually have insufficient number of trainable data. Finally, practical usage to increase diagnostic performance under class imbalance status using the synthesized images were elaborated.

## Materials and methods

### Datasets

The retinal images used in this study were received from the Health Examination Center of the ASAN Medical Center in Seoul, South Korea. Two kinds of dataset were prepared for GAN training. First, a total of 98,561 normal retinal (hereinafter referred to as “normal”) and 20% (26,437 patients) of the other retinal (hereinafter referred to as “uncertain”) patient data were retrieved. Here, we defined a normal subject if both eyes have *normal* keywords in the medical chart and defined the others as uncertain. Retinal images obtained from the second visit for each patient were not included. Of all images from uncertain patient data, only 3.29% have the actual abnormal keyword in the medical chart and the other images from an uncertain patient were regarded as normal or benign normal. The abnormality and their number percentage are shown in Appendix Table 1.

The dataset is in a DICOM (Digital Imaging and Communications in Medicine) format and contains 24-bit RGB retinal images. The original image size is 1536 × 2048 pixels. Header information, except age and sex, was anonymized from the data center. We excluded zoomed retinal images where the peripheries of the major vascular arcades were not shown, and saturated or dark retinal images where the optic discs were not correctly detected. Optic disc detection was performed using a publicly available deep-learning-based method^22^ where not correctly detected images were confirmed by retina specialist (Y. J. Kim). The remaining 98,446 normal and 26,113 uncertain patient data were used to train the GAN. Because most patient data included left and right eye images and some of them have more than two images obtained from follow-up observation, a total of 276,113 images were used for GAN training. In the input data, the average age was 50.3 ± 11.3 years, and 53.5% of the patients were male.

For the evaluations of efficacy of transfer learning, epiretinal membraine (ERM) disease cases shown in Appendix Table 1 were prepared as second dataset. They are total 2671 retinal images from 1975 patient data. The average age was 59.4 ± 8.4 years, and 58.1% of the patients were male. The image preprocessing was done as same as above.

This retrospective study was conducted according to the principles of the Declaration of Helsinki and in accordance with current scientific guidelines. The study protocol was approved by the Internal Review Board (IRB) of Asan Medical Center, University of Ulsan College of Medicine, Seoul, Korea (IRB No. 2019-1373). Because of the retrospective design of the study and the use of de-identified patient data, the review board of IRB of Asan Medical Center, University of Ulsan College of Medicine, Seoul, Korea waived the need for written informed consent.

### GAN training

**Figure 1:**
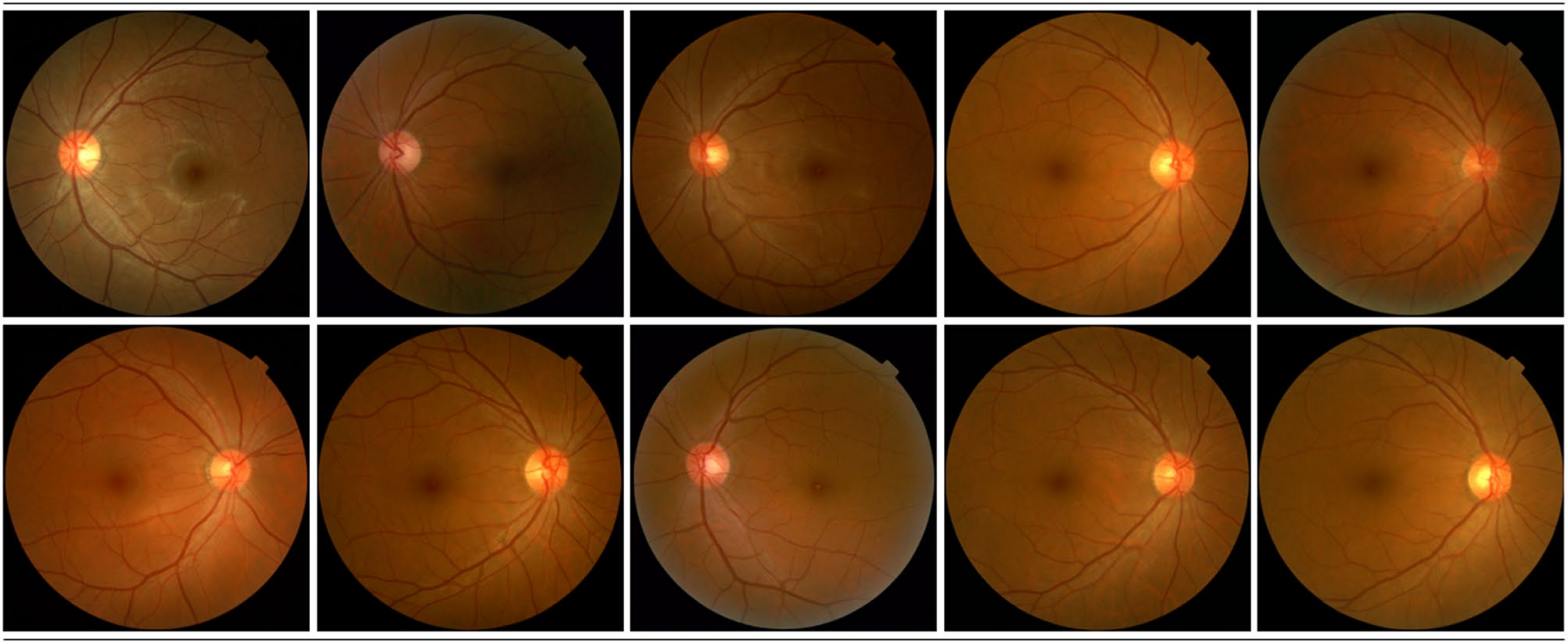
Examples of randomly synthesized images.

For retinal image synthesis, we used StyleGAN^23^, which has been proven successful in image synthesis by various studies^24^. Previously released GAN algorithms trained a mapping function between the input latent vector and target image. However, although it exhibits noteworthy generation power, direct mapping from a latent vector has limitations in changing various visual attributes. On the other hand, StyleGAN sets an intermediate latent space between the latent vector and target image. For the StyleGAN training performed in this study, input images were processed as follows. First, we adjusted the center of each retinal image to be located at the center of a 2-dimensional image plane. Square cropping was then performed on the outer region of the retinal image. Finally, the image was resized to 1024 × 1024 pixels. Two Titan-RTX 24-GB graphics processing units (GPUs) were used, and the learning rate was set at 0.001. Other training parameters were set as default. Source code is from StyleGAN official Tensorflow code. During the training, the resolution of the synthesized images progressively grew until, finally, a 1024 × 1024-sized high-resolution image was synthesized. Examples of the synthesized images are shown in Fig. 1. In Appendix Fig. 1, training curve with frechet Inception distance (FID) score^25^ was shown where the FID is a metric to evaluate statistical similarities between the real and synthesized images. The best model weight was chosen by considering FID score as well as quality of synthesized images examined by retinal specialist (Y. J. Kim).

### Image turing test

To validate whether the synthesized images were realistic, we performed image Turing tests with 40 ophthalmologists, which included residents. For the Turing test, 50 real images were randomly chosen from the whole dataset, and 50 synthesized images were randomly synthesized from the StyleGAN generator with a random latent vector as a seed. These 100 images were then uploaded to a dedicated webpage for the image Turing test. A screenshot of the webpage is shown in Appendix Fig. 2. The webpage displayed each image one by one with no other information. Forty ophthalmologists, comprising of 12 residents, 14 non-retina specialists, and 14 retina specialists, then independently accessed the website to perform the image Turing test. Information on the reader population is outlined in Appendix Table 2. To reduce environmental variability during the Turing test, the images were displayed in the same order for all ophthalmologists, modification of answers was prohibited, and the ratio of real to synthesized images was unknown to the readers. In addition, prior to this test, none of the readers had experience with synthesized retinal images. Under these conditions, all readers successfully finished the image Turing test.

To evaluate the result of the image Turing test, statistical analysis was performed using IBM SPSS software version 23.0. Because the results of the image Turing test were in binary format for each image and the data were correlated with each person and each image, logistic generalized estimating equation (GEE) models were used for evaluating the image Turing test. The ophthalmologists were grouped in two ways: by specialty, i.e., resident, non-retinal specialist, and retinal specialist; and by specialty with consideration for their years of work experience with a 5-year criterion. Through logistic GEE statistics, each class in a group was compared with the resident class based on the following comparison metrics: accuracy, sensitivity, and specificity, where sensitivity refers to the correctness of selecting real images from the entire set of real images. Furthermore, reader examination time per image was recorded to assess performance according to work experience. Because examination time is a continuous value, GEE analysis with identity link was used to compare between classes for both ways of grouping the ophthalmologists.

### Morphological characteristics of synthesized images

**Figure 2:**
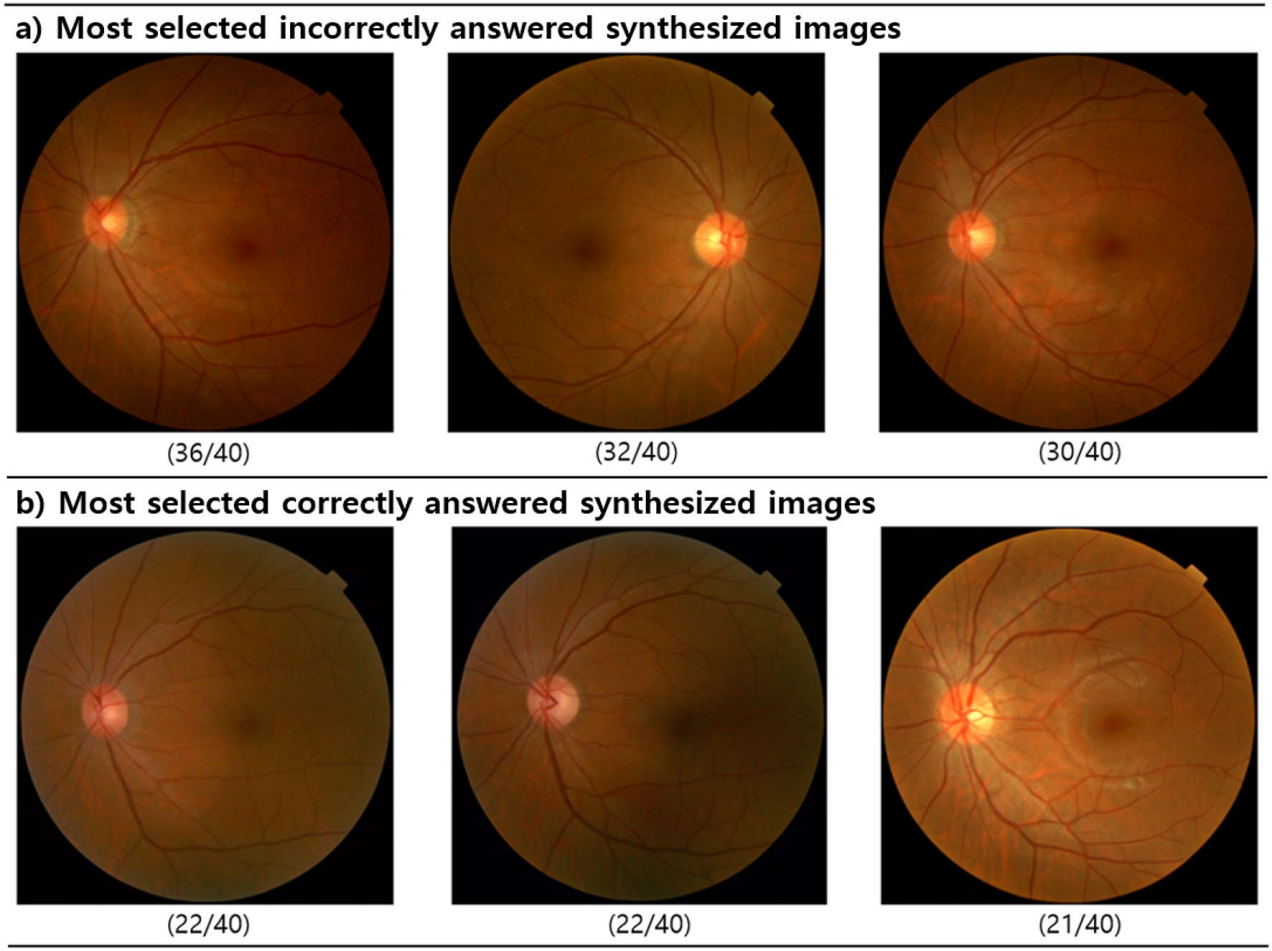
High-resolution synthesized retinal photographs. (**a**) Synthesized retinal images that most ophthalmologists selected as “real” image. (**b**) Synthesized retinal images that most ophthalmologists considered as “synthesized” images. Numbers in parentheses indicate the number of examiners out of 40 ophthalmologists who chose image as “real” or “synthesized.”

The morphological characteristics of synthesized images were examined in comparison with the real retinal images by two retina specialists (Y. N. Kim and Y. J. Kim). Of the 50 synthesized images used for image Turing test, top 3 of the most frequently selected as real images (i.e., incorrectly answered) and top 3 of the most frequently selected as synthesized images (i.e., correctly answered) were analyzed. Top 3 images are shown in Fig. 2. Furthermore, to distinguish the differences between real and synthetic retinal images in greater detail, the anatomical features of three landmarks (optic disc, macular and vascular structures) were manually traced for each of the 50 synthesized images (Appendix Fig. 3).

### Metric evaluation of vessels

Metric evaluations were performed to compare quantitative indices between the real and synthesized images, specifically, comparisons of skeletonized vessel amounts and signal-to-noise ratios (SNR), where vessels and their nearby background regions were defined as signal and noise, respectively. Because both metrics required vessel-segmented images, we first derived vessel segmentation maps using the feature pyramid network (FPN), a deep-learning-based segmentation network^26^. We used a high-resolution fundus (HRF) public database and our data for training the segmentation model. The segmentation performance (area under the curve: AUC 0.80) was comparable to those of the other publicly available segmentation tools. Based on the segmented vessel map, metric evaluation was performed on randomly selected 1,000 real images and randomly synthesized 1,000 synthesized images. For the comparison of vessel amounts, we counted all pixels comprising vessels in each vessel-segmented image.

For SNR measurement, we measured mean signals from vessels in zone B. Zone B is defined as the region between two to three optic-disc diameters away from the optic disc center^27^. The signal and noise of the region were estimated based on the following method.

First, we identified all vessels in zone B using the vessel segmentation map. Five points on each vessel, at even distances, were then selected. At each point, pixel intensities perpendicular to the vessel direction is averaged to define the signal, and a standard deviation of ± 5 neighboring pixels perpendicular to the point but outside of the vessel wall was defined as noise. Appendix Fig. 4 presents in detail the process of SNR calculation. Here, R software (R Foundation for Statistical Computing, Vienna, Austria), version 3.5.3, was used for the statistical analysis, with a significance level of *p* < 0.05.

### Efficacy of the StyleGAN model weight for synthesizing retinal images having specific disease via transfer learning

Unlike the normal and uncertain image cases, retinal images having specific disease is unable to synthesize due to small number of images from original dataset. Nevertheless, this can be overcome through transfer learning the StyleGAN model weight trained for normal and uncertain images even with the small number of images having a specific disease. Through this study, we verified the efficacy of the transfer learning and the usage of synthesized retinal images having ERM disease as an example. Detailed procedures are described as follows and shown in Appendix Fig. 5.

**Figure 3:**
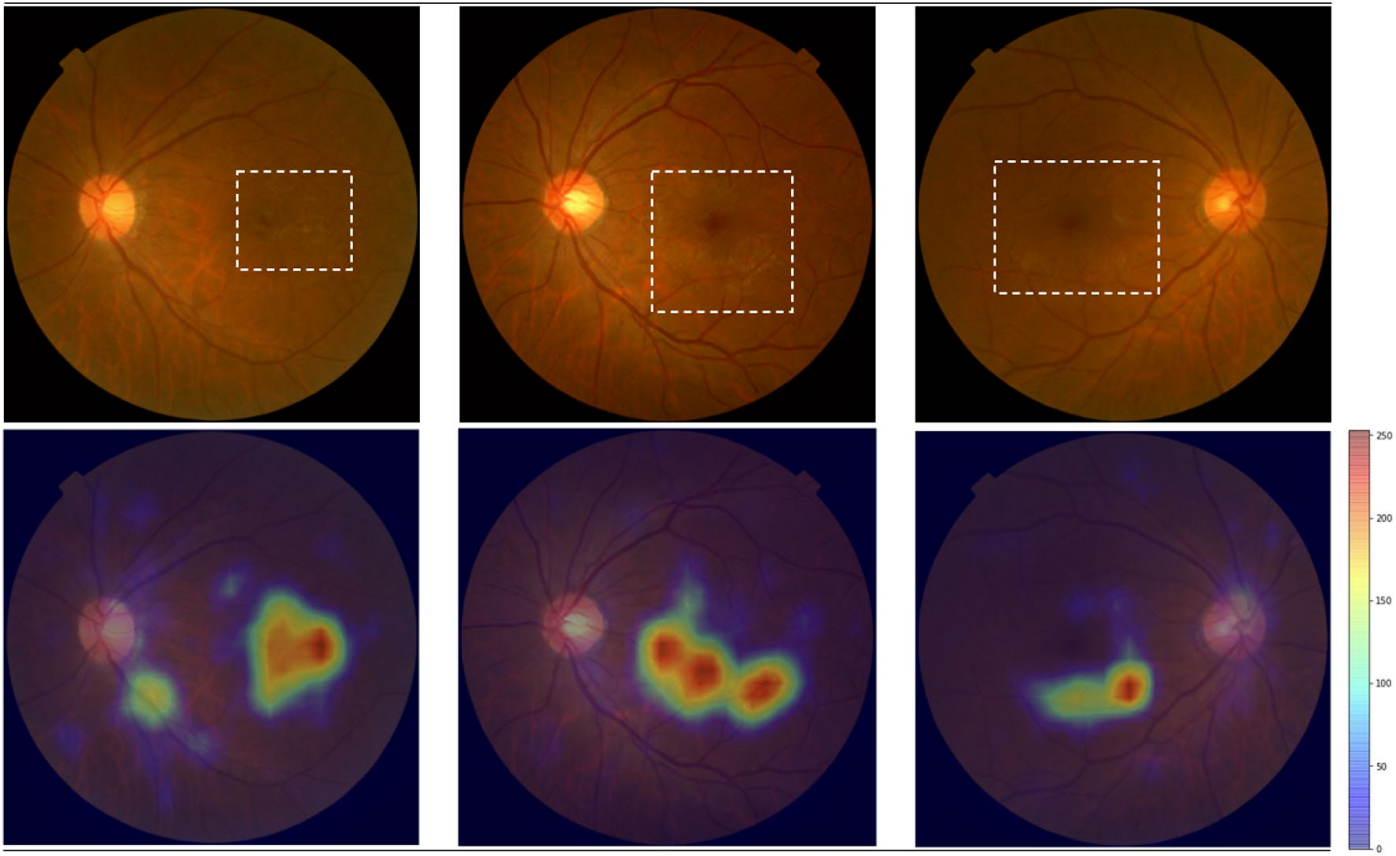
Representative randomly synthesized images demonstrating ERM. Cellophane-like membrane formation at the macula and perifoveal vascular tortuosity are shown in synthetic fundus images. Heatmap derived using Grad-CAM (https://github.com/jacobgil/pytorch-grad-cam) correspond to these characteristic ERM features.

1. Among all dataset collected from Health Examination Center, retinal images were collected by searching *ERM* keywords in the medical chart. Finally, a total of 7476 images were collected.
2. Because keyword searching from medical chart does not guarantee all 7476 images have ERM disease, retinal specialist (Y. J. Kim) examined each image until 600 ERM images and 600 non-ERM images were collected. The residual 6276 images were classified using deep learning model developed at Step 3.
3. To classify residual 6276 images into ERM and non-ERM, deep learning based binary classification model using ResNet152^28^ was developed by training already classified 600 ERM and 600 non-ERM retinal images at Step 2. The dataset was divided into train, valid, and test set with a ratio of 7:1:2. Input image size is 512 × 512 pixels and RGB image is used with geometric image augmentation such as horizontal flip, vertical flip, shift(6.25% of image size), zoom(10% of image size), rotation(± 5degree). Here, augmentation is randomly applied to images at each mini-batch. Learning rate and mini-batch size were 0.0001 and 10, respectively with Adam optimizer^29^. The developed model shows AUC, accuracy, sensitivity, and specificity of 0.986, 0.958, 0.970, and 0.947, respectively.
4. Using the classification model, 6276 images were then classified into 3362 ERM images and 2914 non-ERM images. 3362 ERM images were then divided with ratio of 8:2 where 2671 images were used to develop ERM synthesis model by transfer learning StyleGAN and 691 images were used to develop classification model at Step 6.
5. Using the 2671 ERM images, transfer learning of StyleGAN was performed leveraging the weight from the trained model used for the image Turing test (see Chapter "GAN training"). Specifications of input images and training parameters were the same as the Chapter "GAN training"except for the learning rate which was reduced to 0.0001. The reduced learning rate was applied for fine-tuning the model weight to synthesize ERM features on retinal images. In Appendix Fig. 1, training curve with FID score^25^ was shown. The best model weight was chosen by considering FID score as well as quality of synthesized images examined by retinal specialist (Y. J. Kim). Therefore, retinal specialist (Y. J. Kim) examined the 100 randomly synthesized ERM images and confirmed that characteristics of ERM features, e.g. cellophane-like membrane formation at the macula and perifoveal vascular tortuosity were well synthesized. Examples of synthesized ERM images were shown in upper row of Fig. 3. In bottom row of Fig. 3, heatmaps which is a highlighted attention map representing regions that how the deep learning model trained ERM features was also shown. Here, heatmap is derived using gradient-weighted class activation mapping (Grad-CAM)^30^ with well trained deep learning based classification model using real normal versus real ERM classification model with equal number ratio at Step 6. Retinal specialist (Y. J. Kim) examined the heatmap and concluded that the features are corresponding to the region of ERM features.
6. At this step, efficacy of synthesized ERM images when developing ERM and non-ERM image classification model under imbalanced dataset was evaluated. That is, binary classification was performed by training various number ratios between real normal and real ERM disease (i.e., 1:1, 1:0.5, 1:0.4, 1:0.3, 1:0.2, 1:0.1). Then, compared the classification performance for balanced ratio by adding synthesized ERM images to the imbalanced one. Here, the validation and test set were common to all classification studies. For the training, ERM images were prepared from 691 real images remained at Step 4 and synthesized images generated at Step 5. The non-ERM images were prepared from the dataset for StyleGAN training at Chapter "GAN training". The training parameters including preprocessing method of input images were the same as the classification model developed in Step 3. The detailed combinations for training and characteristics of the dataset were tabulated in Table 1. The training curve for each combination is shown in Appendix Fig. 6.

**Table 1:** Baseline characteristics of study for evaluating classification performance.

Dataset	Normal			ERM			No. ratio (Normal: ERM)
	No. of images	Age, years (SD)	Sex, N (M, F)	No. of images	Age, years (SD)	Sex, N (M, F)	
Train	691	49.79(9.28)	691, 425	691	58.75 (8.06)	401, 490	1:1
	1382	49.73(9.13)	864, 518				1:0.5
				552	58.94(7.93)	314, 238	1:0.4
				414	59.10(7.74)	236, 178	1:0.3
				276	59.37(7.41)	151, 125	1:0.2
				138	59.00(7.55)	77, 61	1:0.1
Valid	197	49.82(9.28)	108, 89	197	59.18(8.79)	110, 87	1:1
Test	396	49.47(8.83)	252, 144	396	59.34(8.57)	225, 171	1:1

## Results

### Image turing test

Table 2 summarizes the results of the realism assessment of all images by 40 readers. The mean accuracy, sensitivity, and specificity of all readers were 54.0 ± 12.3%, 71.1 ± 18.8%, and 36.9 ± 25.5%, respectively. There was no significant difference in accuracy according to the experience levels and subspecialties of the reader groups.

**Table 2:** Image Turing test results. Accuracy, sensitivity, and specificity of each group and each method.

Method	Group ID	Accuracy (95% CI)	Sensitivity (95% CI)	Specificity (95% CI)
Method1	1	0.51 (0.44–0.58)	0.69 (0.56–0.79)	0.33 (0.20–0.50)
	2	0.48 (0.44–0.51)	0.68 (0.50–0.81)	0.28 (0.16–0.45)
	3	0.48 (0.43–0.54)	0.63 (0.51–0.74)	0.33 (0.24–0.44)
	4	0.58 (0.51–0.65)	0.78 (0.68–0.85)	0.38 (0.22–0.57)
	5	0.65 (0.55–0.74)	0.78 (0.63–0.88)	0.52 (0.31–0.71)
Method2	1	0.51 (0.44–0.58)	0.69 (0.56–0.79)	0.33 (0.20–0.50)
	2	0.48 (0.45–0.51)	0.65 (0.55–0.75)	0.31 (0.22–0.40)
	3	0.62 (0.55–0.68)	0.78 (0.69–0.85)	0.45 (0.31–0.60)

Table 3 summarizes the results of a logistic generalized estimating equation (GEE). For both ways of grouping the ophthalmologists, only the resident and most experienced group exhibited statistical significance for accuracy, with a ***P*** of 0.03. On the other hand, sensitivity and specificity did not exhibit statistical significance in any comparison. In addition, examination time for each reader revealed that there were no statistically significant differences. Details on the GEE results in terms of the elapsed examination time are shown in Table 3.

**Table 3:** *P* of Logistic GEE results of each group and each method.

Method	Group ID	Accuracy	Sensitivity	Specificity	Elapsed time
Method1	1	Ref	Ref	Ref	Ref
	2	0.44	0.92	0.62	0.21
	3	0.53	0.51	0.97	0.85
	4	0.18	0.20	0.71	0.11
	5	0.03	0.31	0.18	0.48
Method2	1	ref	ref	ref	ref
	2	0.45	0.67	0.75	0.39
	3	0.03	0.18	0.29	0.18

### Morphological analysis of landmarks in retinal images

Figure 2 shows the most frequently selected retinal images. According to reviews of the most frequently incorrectly selected synthesized images, the overall color, choroidal texture through the retina, and shapes of the optic cup and disc were similar to those of real retinal images. On the other hand, in the most frequently correctly selected synthesized images, dark and uneven illumination was identified. The optic discs were faint in color, and the integrities of the retinal vascular contours were interrupted.

Appendix Fig. 3 examined more detailed features of synthesized images. Inside the optic cup, the sieve-like structures of lamina cribrosa were accentuated with flame-shaped markings. Moreover, vessels transversely crossing the optic disc, which did not conform to the normal vascularity originating from an optic cup and were unlikely to be normal central vessel trunks, were observed (Appendix Fig. 3a). With regard to the macular structure (Appendix Fig. 3b), atypical foveal reflections irrelevant to the position of the main vascular arcade were recognized. In addition to its uneven surrounding illumination, the macula was characterized by either an absence or excessive macular pigmentation compared to that in normal retinal images. Lastly, the most frequently present features were abnormal vascular configurations of the peripheral retinal vessels (Appendix Fig. 3c). The vessels in the synthetic images exhibited ill-defined vascular margins, such as beading vessels and orphan vessels having bizarre vascular courses of unclear proximal vascular origins.

### Metric evaluation of vessels

The average vessel amounts in the real and synthesized images were 1.18 × 10^5^ ± 2.0 × 10^4^ and 1.14 × 10^5^ ± 2.8 × 10^4^ pixels, respectively. Unpaired t-tests yielded a *P* < 0.001 with a mean difference of 4,512 pixels. Because the mean difference was only 0.43% of the image size and 3.9% of the average vessel amount of both real and synthesized images, we concluded that the difference was clinically negligible. The average SNRs of the real and synthesized images were 1.69 ± 0.10 and 1.7 ± 1.09. Unpaired t-tests were conducted, and a *P* < 0.001 was found, but the mean difference in SNRs between real and synthesized images was only 0.03 dB, which was 1.5% of average SNRs for both. We also concluded that the mean difference of 0.03 dB is insignificant. Standardized mean difference, also called Cohen’s d, between the real and synthesized groups was 0.18 for vessel amount and 0.19 for SNRs^31,32^. Cohen’s d values smaller than 0.2 in our measurements additionally supported the conclusion that the differences between the real and synthesized images are clinically insignificant^33^.

### Efficacy of the StyleGAN model weight for synthesizing retinal images having specific disease via transfer learning

Table 4 shows the classification performance for various ratios between the real normal and real ERM images. Each ratio has two kinds of results except study for the equal ratio shown at the first row of the table. The upper one shows model results trained by only imbalanced real datasets and the bottom one shows model results trained by balanced datasets after adding synthesized ERM images. Although the best performance was shown for equal ratio from the real-only dataset, all studies by adding synthesized ERM images on the imbalanced real dataset outperform the imbalanced one. Especially, the most extremely imbalanced case (i.e. number ratio of 1:0.1) shows an AUC increment of 23.7% (from 0.735 to 0.909) after balancing the ratio using synthesized ERM images. The training curves shown in Appendix Fig. 6 indicate the overfitting is getting higher at larger imbalanced dataset. In addition, balanced dataset using synthesized images converges much faster than imbalanced dataset at training curve.

**Table 4:** Classification performance for various ratios between normal and ERM. AUC, accuracy, sensitivity, and specificity were shown for each ratio with and without adding synthesized ERM images.

No. ratio of real dataset (Normal:ERM)	Add synthesized ERM*	AUC	Accuracy	Sensitivity	Specificity
1:1	No	0.994	0.971	0.965	0.977
1:0.5	No	0.988	0.963	0.955	0.972
	Yes	0.989	0.970	0.957	0.982
1:0.4	No	0.983	0.943	0.909	0.977
	Yes	0.994	0.970	0.942	0.997
1:0.3	No	0.984	0.905	0.826	0.985
	Yes	0.987	0.968	0.947	0.990
1:0.2	No	0.943	0.874	0.843	0.904
	Yes	0.966	0.914	0.904	0.924
1:0.1	No	0.735	0.559	0.174	0.944
	Yes	0.909	0.739	0.508	0.970

*Yes means adding synthesized ERM for balancing number ratio with normal dataset.

## Discussion

Using the GAN approach, we were able to successfully synthesize synthetic retinal images. Ophthalmologists were not able to distinguish between real images and synthesized images with a random probability of 54.0 ± 12.3%. The mean sensitivity was higher than the mean specificity, which indicates that the synthesized images appeared mostly real and that it was difficult to discriminate whether the synthesized images were real or not. In addition, metric analyses revealed no significant differences between the real and synthetic images. Even though several aspects of the optic discs, maculae, and vascular structures could be discriminated as synthetic properties when readers with advanced experience evaluated the images carefully and attentively, the synthetic images were, overall, highly acceptable.

Our powerful image synthesis capabilities may have originated from a large number of high-resolution datasets compared to those in previous studies. Burlina et al.^16^ used PGGAN to synthesize retinal images having AMD with an image size of 512 × 512 pixels. Two retinal specialists distinguished the real and synthesized retinal images of various AMD stages and concluded that the AMD features were well synthesized and have the potential for education usage and image augmentation. Zhao et al.^20^ trained a GAN network to synthesize retinal images, using a vessel annotation map as an input; however, this approach had limitations, specifically with synthesizing uniform vascular structures in the uniform matter and the locations, shapes, and boundaries of landmarks not being clear because of a small dataset. To reduce this problem of the unclear synthesis of landmarks, Yu et al.^19^ attempted to synthesize retinal images from combined landmarks, such as vessel maps, optic discs, and optic cups. Although this approach resulted in clearer landmark synthesis, the vascular structures remained unchanged, and the spatial resolution did not reach 1024 × 1024 pixels. Recently, Andreini et al.^14^ suggested a two-step approach to synthesizing a variety of vascular structures: the synthesis of vascular structures using Progressive Growing of GAN (PGGAN^34^), with a publicly available small dataset as input; and image translation from vascular structures to retinal images using Pix2PixHD^35^. This approach enabled the researchers to synthesize a variety of vessel maps and retinal images characterized by a variety of vascular structures. However, the landmarks and boundaries of the retinal images were unclear, and disconnected vessels were also synthesized. The researchers speculated that the low-resolution vascular structures in the synthesized images were due to the limited number of available training samples, a problem that can be solved via data augmentation, semantic label–maps, or the addition of new layers to the networks. In our study, the large dataset of real, high-resolution images improved the resolutions of the synthetic images and contributed to the real and synthetic images not having any significant differences between them.

**Figure 4:**
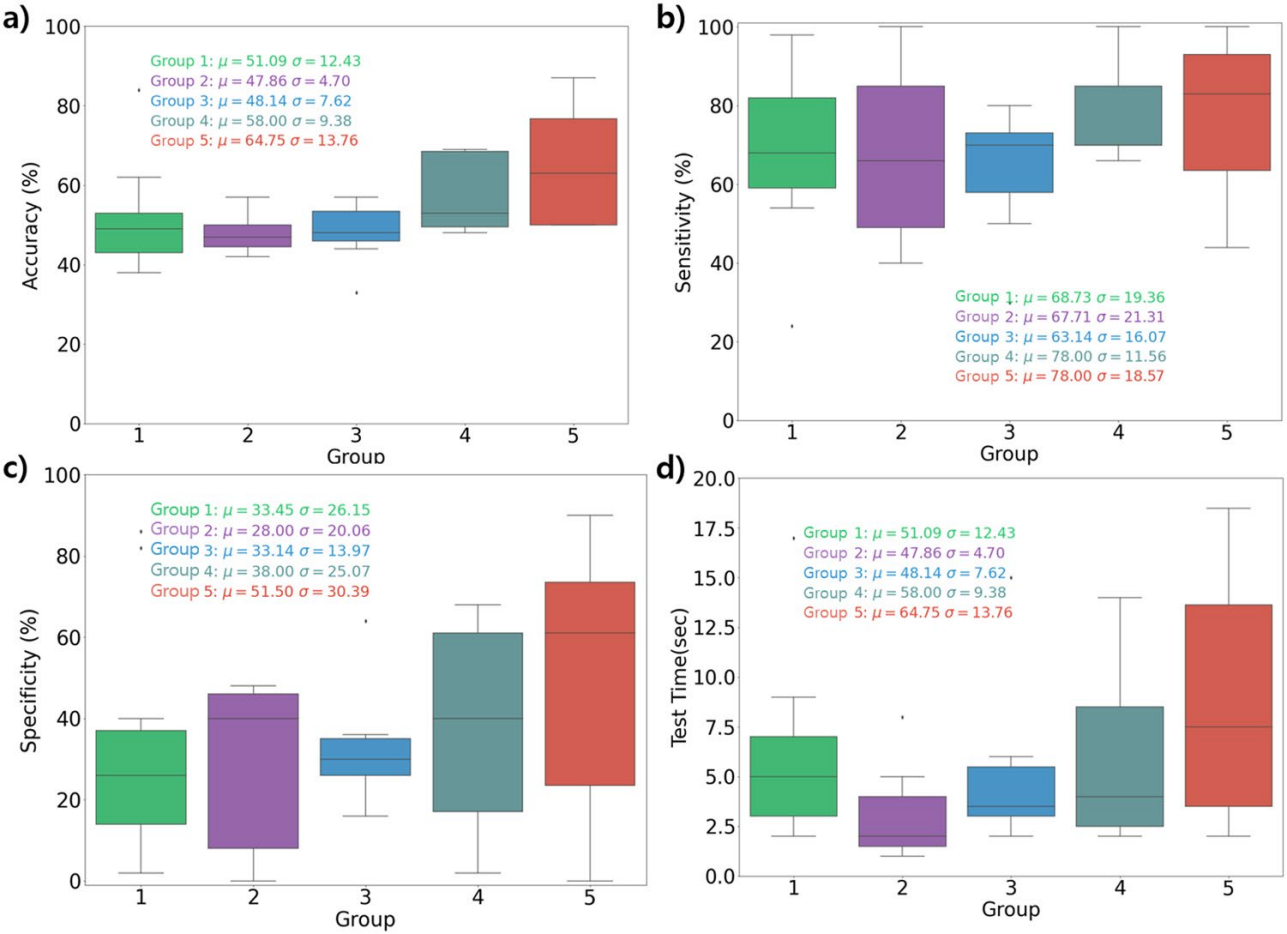
Group-averaged performance of image Turing test: (**a**) accuracy, (**b**) sensitivity, (**c**) specificity, (**d**) elapsed time for each image. Numerical values of average estimates and their standard deviations are also shown.

Nevertheless, an interesting result of our study is that the evaluated metrics, i.e., accuracy, sensitivity, specificity, and image examination time, increased with work experience (see Fig. 4), although there exists no considerable statistical significance. This trend suggests that the more experienced ophthalmologists examined more landmarks and overall features on the retinal images to determine whether the images were real or synthesized and thus required more time on each image and resulted in better performance. Visual inspections by two retinal specialists (Y. N. Kim and Y. J. Kim) revealed somewhat ambiguously described optic disc contours and related central vessel trunks, inappropriately pigmented maculae, and discontinuities of peripheral retinal vessels from the synthesized images. The common characteristics of these indicated points are that these are microstructures associated with vessels in the retina; the differences in the resolutions of the vascular contours between the real and synthesized images are considered the key for the examiners in image Turing test. If these inspection points are used to train image-generation models to select high-quality retinal images through supervised learning, more sophisticated well-fitted GAN images may be created in future research studies.

Metric analyses of the vessels showed that there were insignificant differences between the real and synthesized images in terms of vessel amount and SNRs. This result indicates that the synthesized images have the potential to be used on vessels themselves or in vessel-related feature analysis, such as in developing vessel segmentation algorithms for predicting DR, AMD, or other retinal vascular diseases, and even for predicting cardiovascular risk.

Moreover, leveraging the model weight trained for mostly normal retinal images, successful transfer learning using a small number of ERM images was performed. The evaluations were done in two ways. One is the visual examination by a retina specialist (Y. J. Kim) and the other is comparing classification model performance with and without adding synthesized ERM images for balancing the imbalanced real normal and ERM dataset. The classification performance was increased after balancing the dataset by adding synthesized ERM images. This indicates that synthesized images contain ERM features. A similar approach was performed by Lim et al.^18^. They synthesized retinal images having DR disease with an image size of 512 × 512 pixels. Then, compared augmentation efficacy with and without adding synthesized retinal images. As a result, the classification performance (i.e., normal versus DR) outperforms after adding the synthesized image.

The succession of the classification task represents the following meaningful point of view. In the medical field, an imbalanced dataset is common causing misclassification for developing deep learning-based models. Using only a traditional augmentation such as geometric or intensity transformations has a limitation to increase model performance. Thus, various generative models including GAN should be utilized if possible. However, a generative model needs at least several thousand of images to synthesize high-quality images. In our case, the number of ERM images is only less than 3000 images and thus expect poor synthesis performance if one trains the GAN model from the model weight having no prior feature knowledge of the retinal images. To overcome the problem, transfer learning was achieved using model weight trained from a few hundred thousand retinal images which comprised of mostly normal patients. Our approach also shows a potential that any disease features would be well synthesized using only a few thousand of specific disease images if model weight was initially well trained from a sufficiently large number of normal datasets.

The GAN introduced through this study is a powerful tool for synthesizing images and thus has great potential to be used in various aspects of medical image analysis. First, GAN is able to avoid the legal and ethical problems associated with medical data containing the personal information of patients^36^.

Second, the performance of deep-learning models can be improved via data augmentation^37^. Although traditional data augmentation techniques such as rotation, geometric transformation, and blurring exist, these transforms do not change much from the original data, thus limiting these models from learning a variety of features and variations in data for deep learning^38^. On the other hand, research on GAN augmentation verified that, as a result of the augmentation, deep-learning models achieved significant improvements^39^. The efficacy of GAN as an augmentation tool was also verified in our study. Data imbalance results in a model be a bias, leading to better predictions for classes with larger amounts of data. Because data imbalance can be caused by a variety of reasons, such as incidence of specific disease and data collection difficulties, synthesizing images for classes with insufficient number of data may be a necessary process for reducing the model bias.

However, our study has several limitations. First, this retrospective study have biased data that were mostly controlled by the same data acquisition condition and patient population. Second, the image Turing test and metric evaluations of vessels were focused only on normal retinal images. Although the ratio of normal and uncertain patients was 8:2, the abnormality is very minor since the data was provided from the Health Examination Center. One thousand retinal images were randomly synthesized and examined by a retinal specialist (Y. J. Kim) and confirmed that all the synthesized images have normal-like features. Nevertheless, ERM disease images were collected and transferred for GAN training. The synthesized ERM disease images were confirmed by retinal specialist that cellophane-like membrane formation at the macula and perifoveal vascular tortuosity were clearly generated. In addition, the disease images show efficacy for increasing classification performance by augmenting it to training dataset. Third, regarding the quality of the synthesized images, visual inspections by two retinal specialists revealed that the GAN-synthesized images required improvements, specifically in terms of understanding the anatomies of optic discs and vascularities, expressing the three-dimensional global structures of maculae, and describing small vascular margins in detail. Forth, this work does not include comparison studies between various kinds of generative models including GAN (e.g. StyleGAN3). Further studies for comparing among generative models should be performed for choosing the best model for clinical application. Fifth, this work does not compare with other approaches overcoming imbalanced dataset such as oversampling, undersampling, or weighted cross-entropy loss. Comparing with this work which trained distribution of training dataset and synthesize unlimited number of images within the data distribution, those approaches have limitations to train the various characteristics of image features. To evaluate the efficacy of synthesized images, further studies are needed to compare robustness to external dataset where domain adaptation or standardization is one of the hottest issues in machine learning^40^.

In conclusion, we trained GAN for synthesizing realistic high-resolution retinal images using 1024 $\times$ 1024 pixels of real retinal images. According to image Turing tests and metric analyses, the synthesized images were difficult to discriminable from real images. In addition, leveraging the model weight which knows features of normal retinal images, transfer learning was successfully performed for ERM images and quality evaluations through visual examinations and downstream tasks show the efficacy of synthesized retinal images as a GAN based augmentation tool. We believe that these synthesized images are novel and have significant potential for medical image analysis, specifically in protecting patient privacy and enhancing physician training as well as developing a deep learning model.

## Supplementary Information

Supplementary Information.

## Data Availability

Real datasets used in this study are not publicly available because of restrictions in the data-sharing agreements with the data sources. Synthesized datasets in the presented study are available from the corresponding author on reasonable request.
