# Supplementary material for: Synthesizing realistic high-resolution retina image by style-based generative adversarial network and its utilization
Source: Sci Rep. 2022 Oct 15;12:17307. doi: 10.1038/s41598-022-20698-3 (PMC9569369; doi:10.1038/s41598-022-20698-3)
Supplement: Supplementary file 1 — Supplementary Information. [file 41598_2022_20698_MOESM1_ESM.pdf]

Synthesizing Realistic High-resolution Retina Image by Style-based Generative Adversarial Network and its Utilization

Mingyu Kim, PhD; You Na Kim, MD; Miso Jang, MD; Jeongeun Hwang, PhD; Hong-Kyu Kim, MD; Sang Chul Yoon, MD; Yoon Jeon Kim, MD; and Namkug Kim[a,f], PhD

Appendix

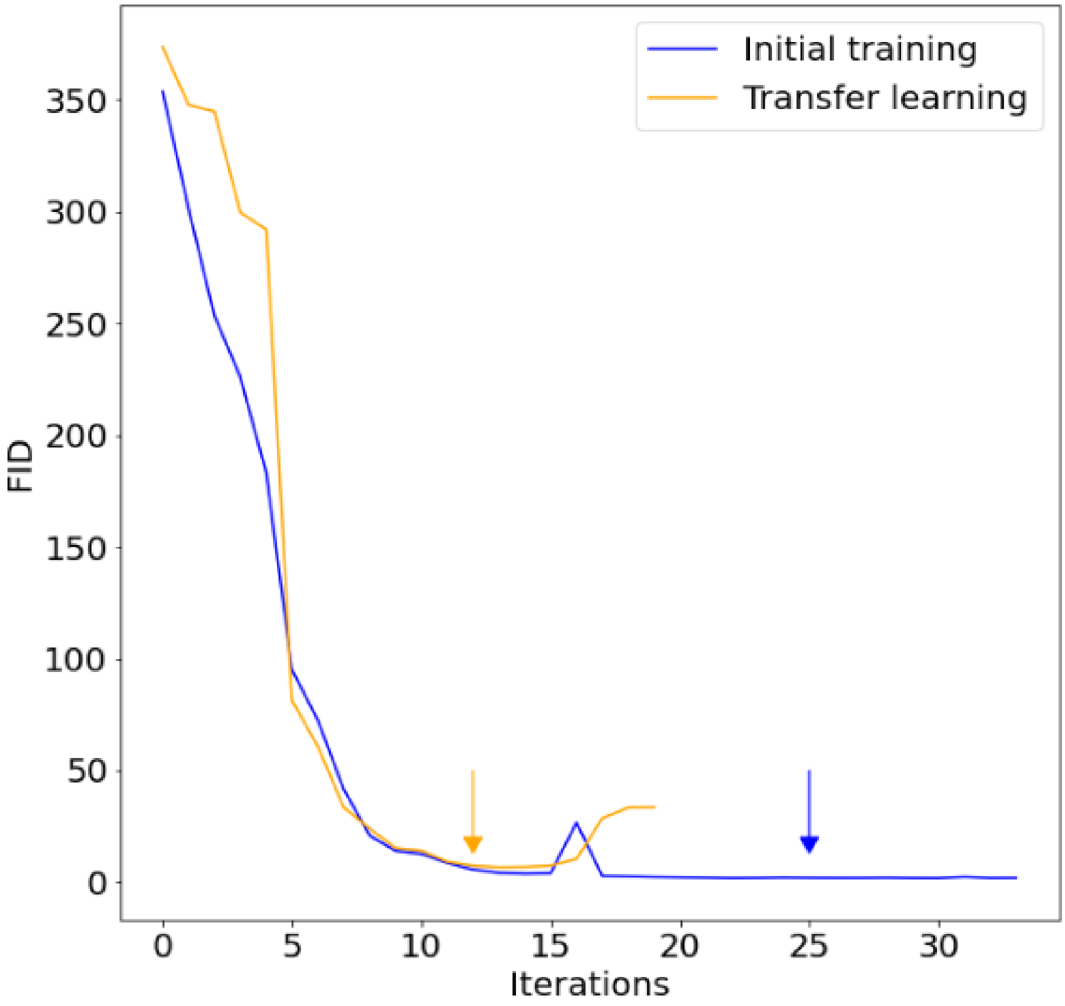

Appendix Figure 1. Training curve with frechet Inception distance (FID) score. Initial training represents StyleGAN training using normal and uncertain retinal images. Transfer learning represents StyleGAN training by transfer learning weight from Initial training using retinal images having ERM disease. Arrow indicates best weight chosen for each training.

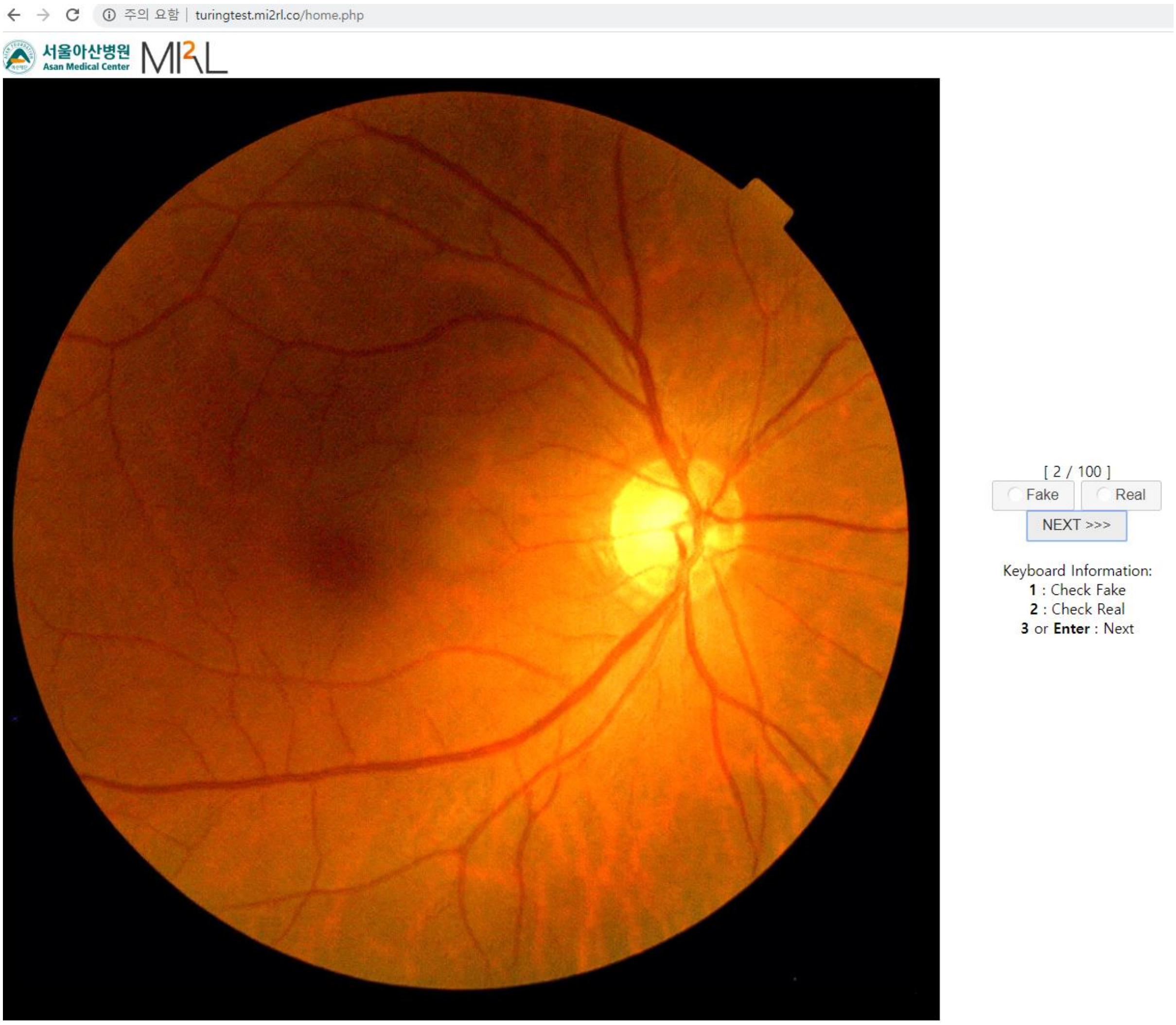

Appendix Figure 2. Example screenshot of dedicated webpage for image Turing test.

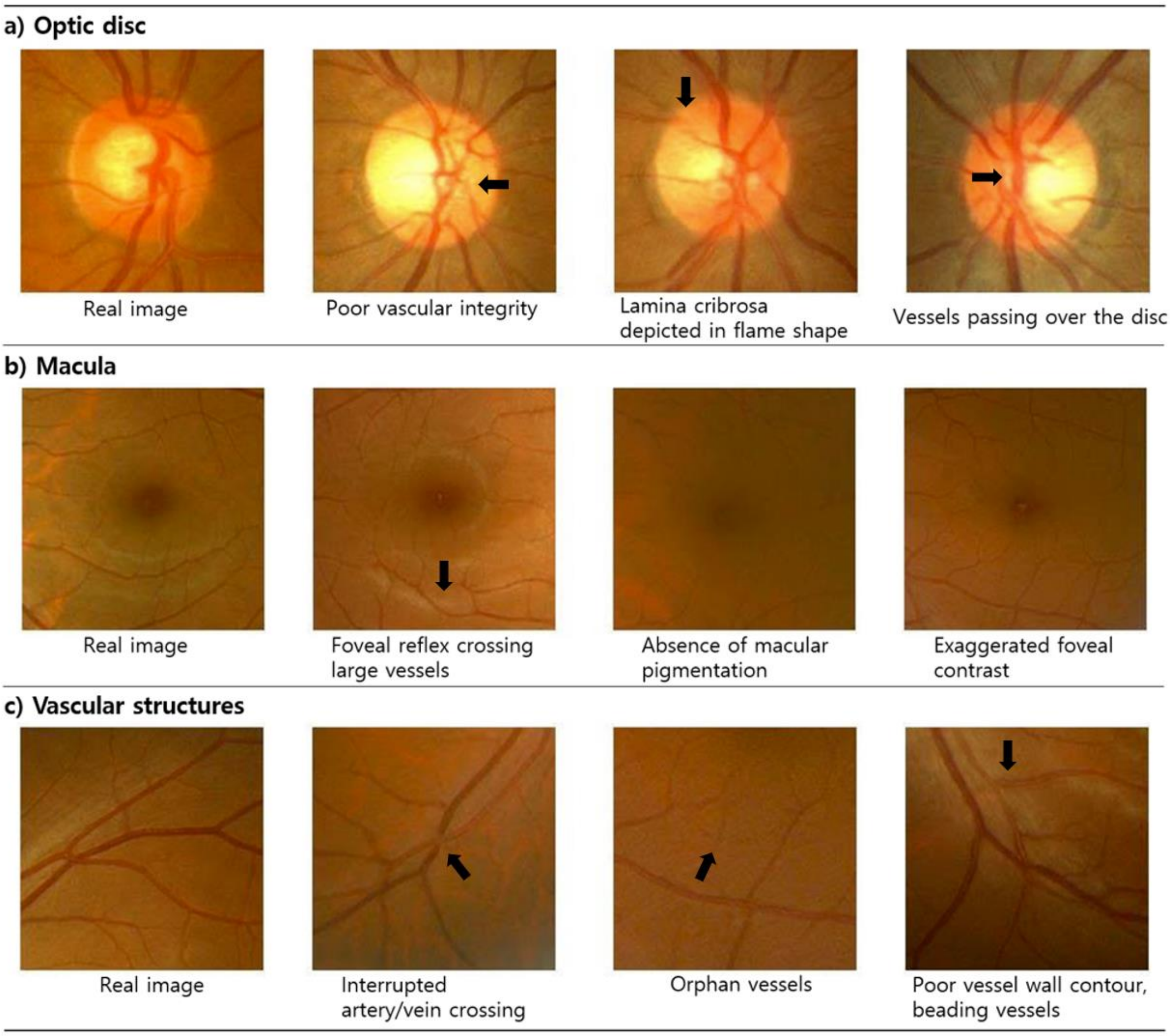

Appendix Figure 3. Comparisons of landmarks that influence selection of "real" and "synthesized" images by ophthalmologists. From top to bottom, detailed images of a) optic discs and b) macular and c) vascular structures to highlight differences between "real" and "synthesized" images. Black arrows indicate regions of inaccurately constructed structures.

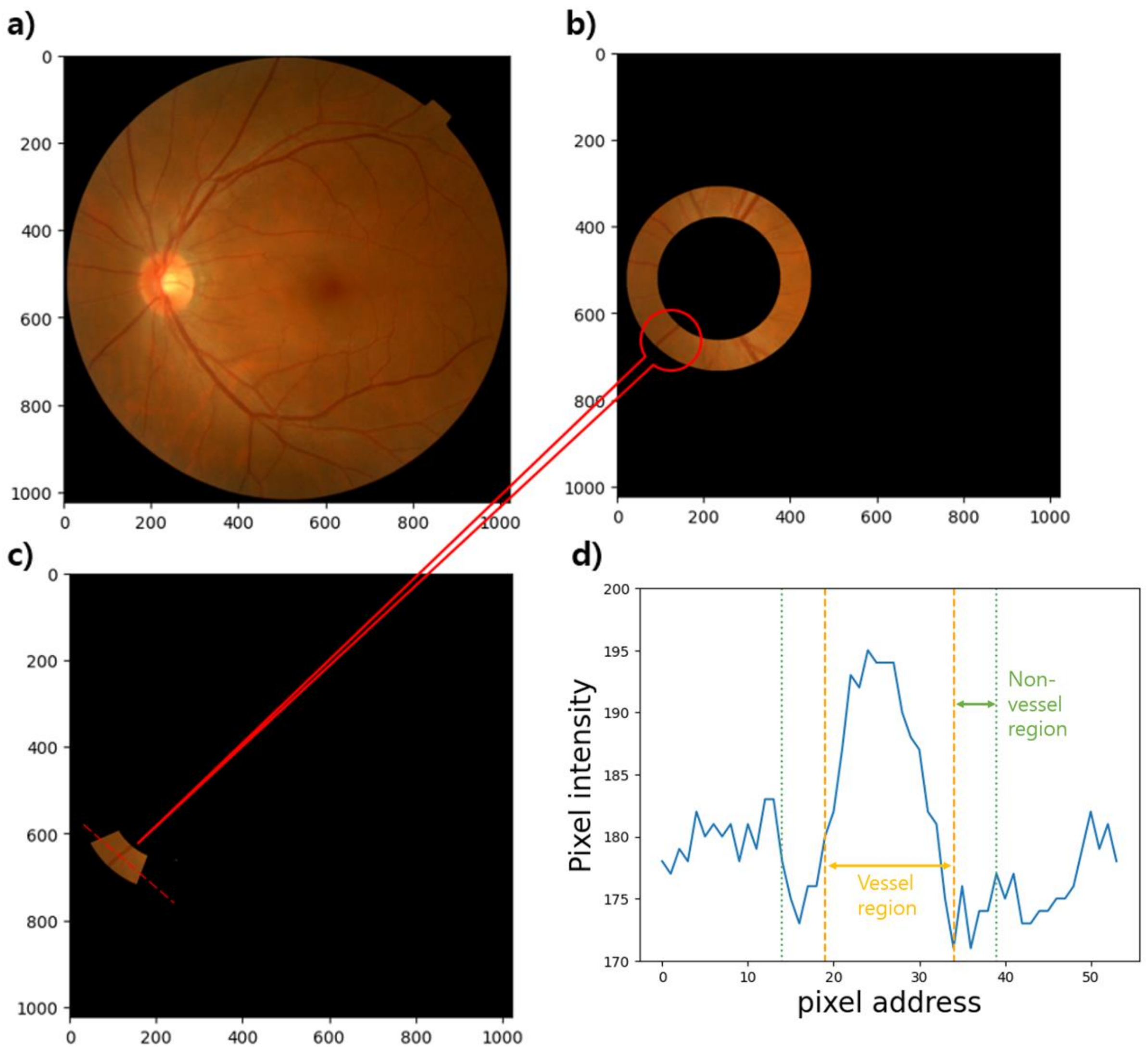

Appendix Figure 4. Flowchart of SNR estimation: a) original image, b) zone B region image. Other regions were masked. c) Example of vessel in zone B region and its perpendicular virtual line. d) Pixel intensity profile of perpendicular virtual line.

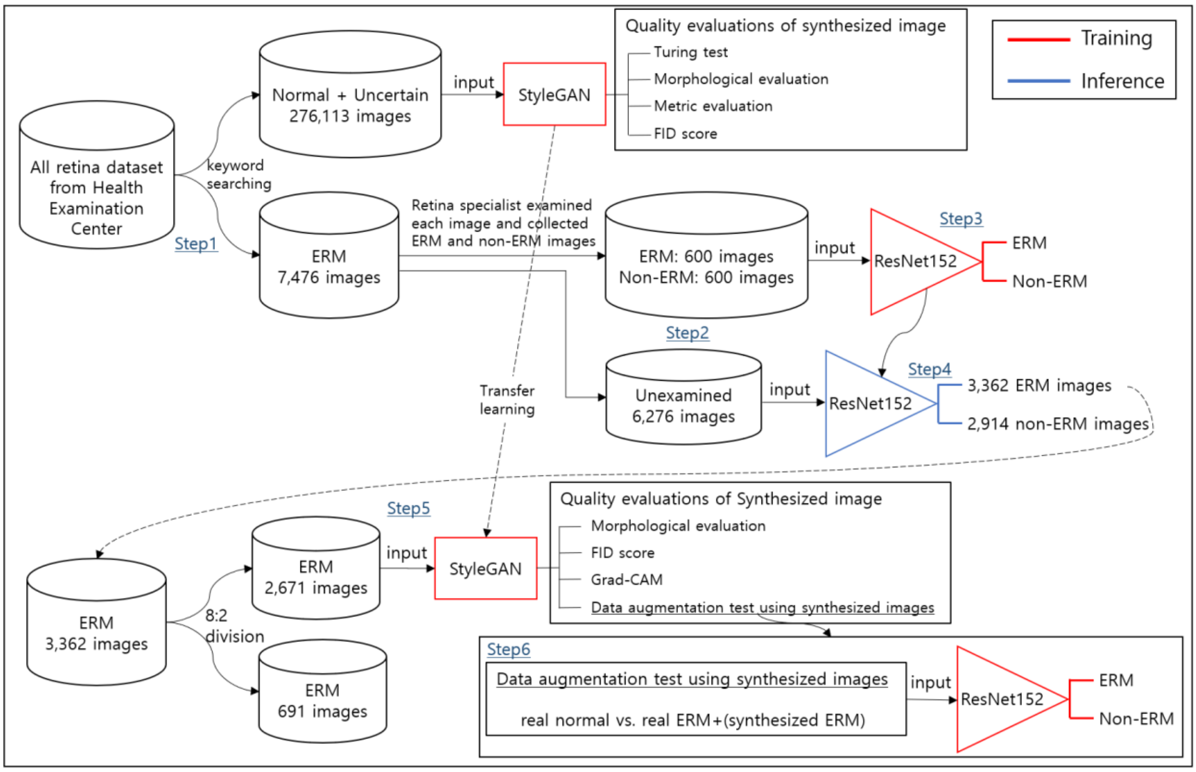

Appendix Figure 5. Flowchart of transfer learning and synthesized image usage from Step1 through Step6 described in Chapter 2.6.

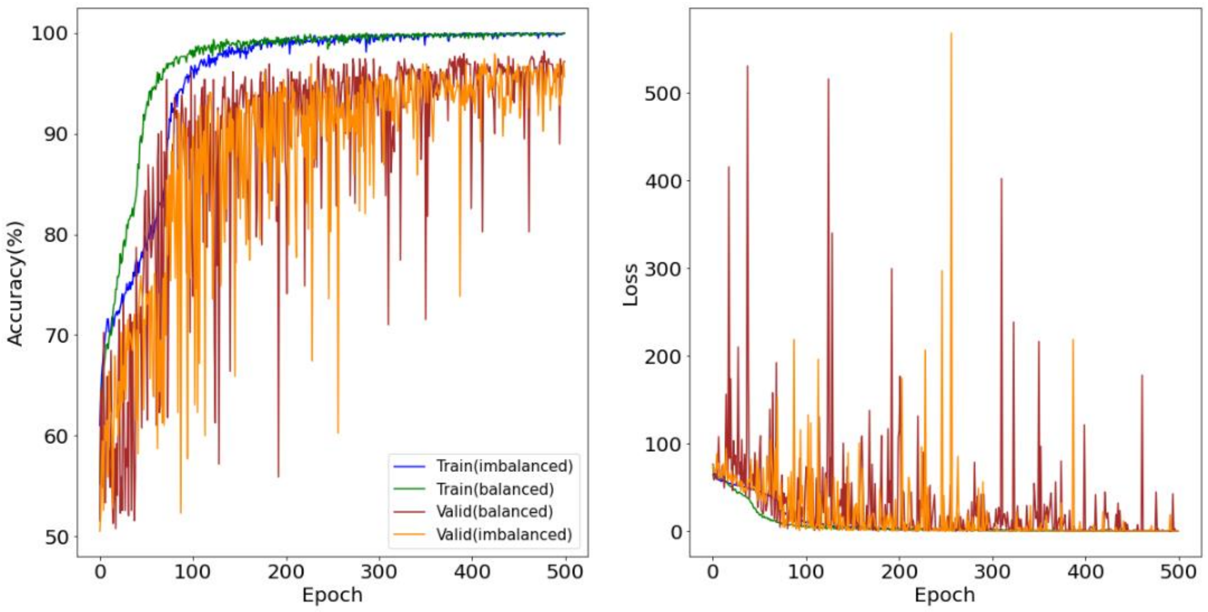

Appendix Figure 6a. Training curve of ERM and non-ERM classification model with balanced and imbalanced (number ratio of ERM vs. non-ERM equal 1:0.5) dataset.

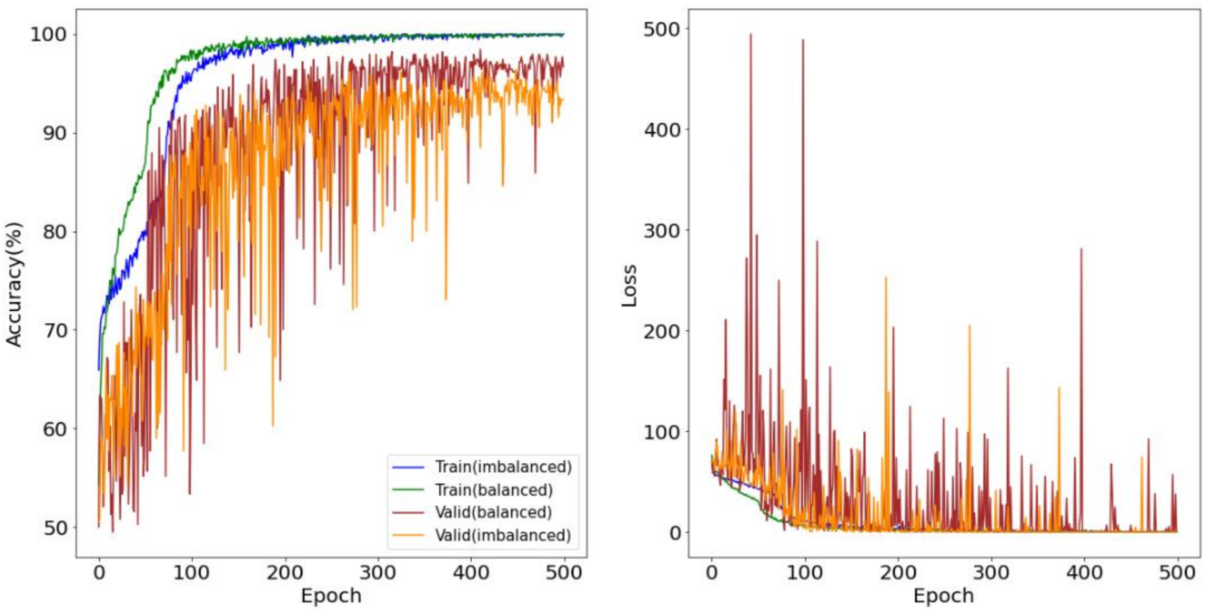

Appendix Figure 6b. Training curve of ERM and non-ERM classification model with balanced and imbalanced (number ratio of ERM vs. non-ERM equal 1:0.4) dataset.

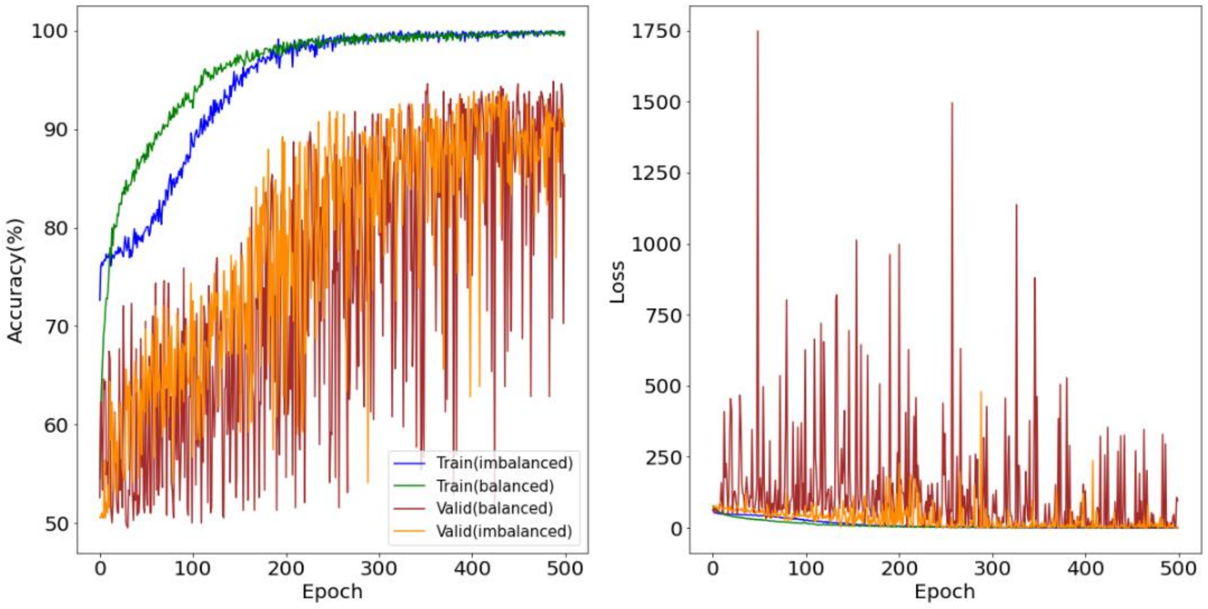

Appendix Figure 6c. Training curve of ERM and non-ERM classification model with balanced and imbalanced (number ratio of ERM vs. non-ERM equal 1:0.3) dataset.

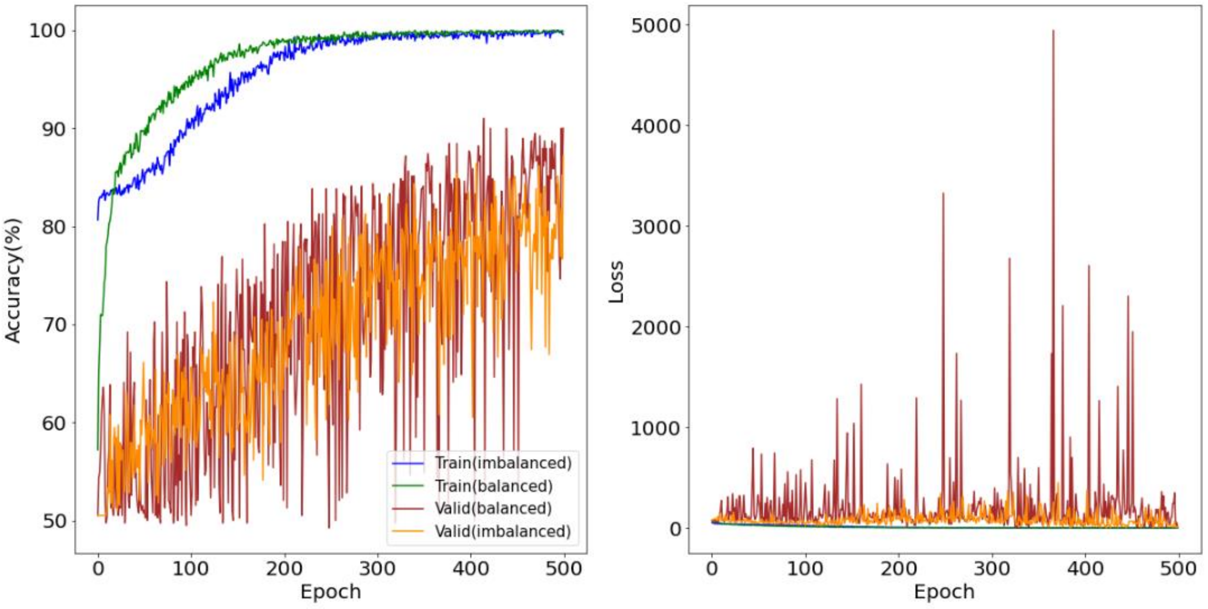

Appendix Figure 6d. Training curve of ERM and non-ERM classification model with balanced and imbalanced (number ratio of ERM vs. non-ERM equal 1:0.2) dataset.

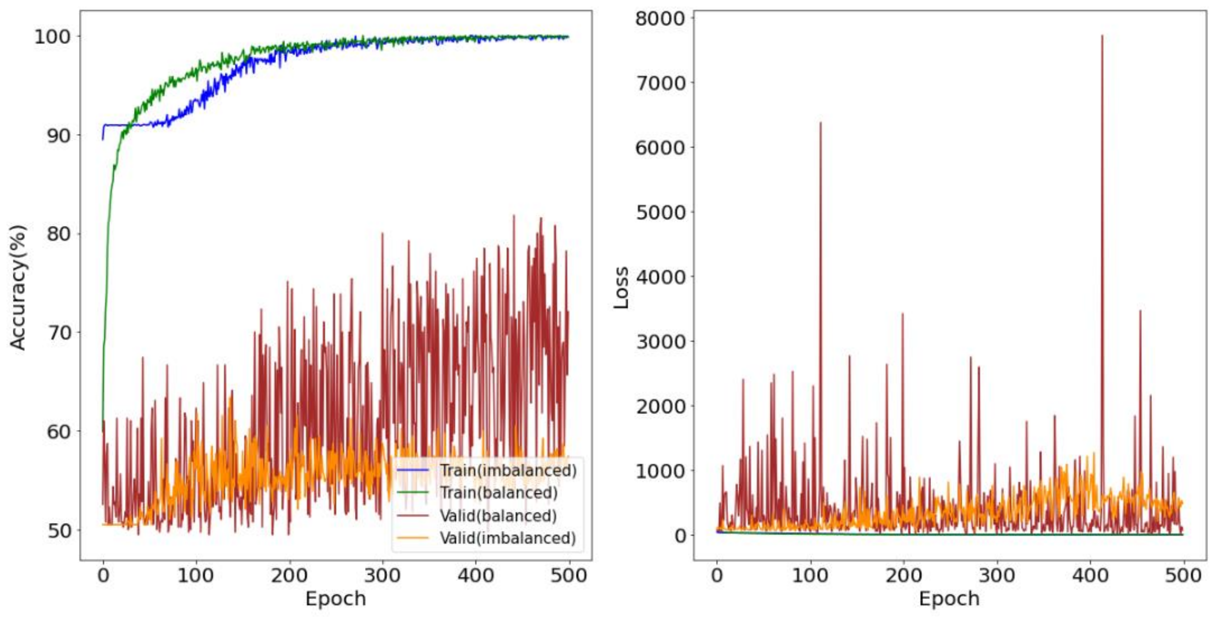

Appendix Figure 6e. Training curve of ERM and non-ERM classification model with balanced and imbalanced (number ratio of ERM vs. non-ERM equal 1:0.1) dataset.

Appendix Table 1. Top 10 disease populations and their percentage among all uncertain images used for StyleGAN training.

No.	Name	Number of images	Percentage of all uncertain images(%)
1	ERM	3222	1.167
2	Glaucomatous disc	1106	0.401
3	Simple retinal hemorrhage	987	0.357
4	Chorioretinal scar	937	0.339
5	Diabetic retinopathy	852	0.309
6	Asteroid hyalosis	377	0.137
7	Myelinated nerve fiber	350	0.127
8	Hard exudates	348	0.126
9	Cotton wool patch	270	0.098
10	Age-related macular degeneration	10	0.004

Appendix Table 2. Population of participants for image Turing test

Group	Years of experience	Number	Group ID (Method1)	Group ID (Method2)
**Residents**	-	12	1	1
**Non-retina specialist**	< 5 years	7	2	2
**Non-retina specialist**	> 5 years	7	3	
**Retina specialist**	< 5 years	7	4	3
**Retina specialist**	> 5 years	7	5	

Method1 = division of ophthalmologists based on specialty and years of experience

Method2 = division of ophthalmologists based on specialty
